# Supplementary material for: Associations of Dietary Fats with All-Cause Mortality and Cardiovascular Disease Mortality among Patients with Cardiometabolic Disease
Source: Nutrients. 2022 Aug 31;14(17):3608. doi: 10.3390/nu14173608 (PMC9460477; doi:10.3390/nu14173608)
Supplement: Supplementary file 1 [file nutrients-14-03608-s001.zip › nutrients-1858208-supplementary.pdf]

Table S1. Baseline characteristics of patients with cardiometabolic diseases in NHANES III and NHANES 1999-2014 according to the tertiles of energy percentage from ω-6 PUFA and ω-3 PUFA [a]

**Characteristics**	**Tertile of ω-6 PUFA intake**			**Tertile of ω-3 PUFA intake**		
	T1	T2	T3	T1	T2	T3
Participants (*n*)	2845	2846	2846	2845	2845	2845
Fatty acid intake (% of energy)	3.92	6.26	9.58	0.40	0.65	1.12
Male (%)	46.88	48.96	49.75	45.60	49.70	50.31
Age (years)	61.42	61.75	60.80	60.71	61.49	61.75
Race (%)						
Non-Hispanic White	70.75	70.45	72.93	70.63	71.26	72.31
Non-Hispanic Black	12.44	14.48	13.75	12.95	14.61	13.07
Mexican American	6.07	6.77	5.63	7.33	5.72	5.35
Others	10.74	8.30	7.70	9.08	8.41	9.27
Education level (%)						
Less than high school	37.46	33.68	27.40	38.12	32.11	28.17
High school or equivalent	25.37	29.27	28.77	27.19	28.60	27.56
College or above	37.17	37.06	43.83	34.69	39.29	44.27

Family income-poverty ratio level (%)						
< 1.3	28.90	25.50	21.67	29.27	25.21	21.55
1.3 - 2.4	31.09	33.36	30.42	32.17	33.25	29.37
≥ 2.4	40.00	41.15	47.91	38.57	41.54	49.09
BMI group (%)						
< 18.5	0.87	1.88	0.69	1.19	1.51	0.69
18.5 -< 25	21.96	20.68	17.16	20.65	20.80	18.31
25 -< 30	32.68	30.01	31.34	33.26	29.76	31.06
≥ 30	44.49	47.43	50.81	44.90	47.93	49.95
Smoking status (%)						
Never smoker	41.76	42.47	42.90	41.12	42.84	43.17
Former smoker	37.27	37.16	39.16	38.09	36.31	39.21
Current smoker	20.97	20.38	17.94	20.79	20.85	17.61
Alcohol drinking (%)						
Non-drinker	79.26	79.10	83.30	79.56	80.08	82.13
Low to moderate drinker	11.46	14.50	10.84	11.61	12.57	12.48
Heavy drinker	9.28	6.40	5.85	8.83	7.35	5.39

Physical activity (%)						
Inactive	38.41	39.97	43.13	38.12	40.13	43.32
Insufficiently active	35.01	33.92	31.93	34.02	34.96	31.87
Active	26.58	26.11	24.94	27.86	24.91	24.81
Self-reported health status (%)						
Poor to fair	46.95	40.66	42.04	45.93	42.78	41.08
Good	32.37	38.04	37.82	32.98	35.70	39.43
Very good/excellent	20.68	21.30	20.14	21.09	21.52	19.49
Self-reported chronic diseases (%)						
Hypertension	60.56	63.42	64.53	61.12	63.21	64.17
Hyperlipemia	64.72	70.23	71.59	65.69	70.29	70.53
Cancer	16.44	19.44	18.23	17.14	17.59	19.28
Total energy intake (kcal/d)	1689.80	1802.57	1933.60	1710.30	1847.73	1870.75
Protein intake (% of energy)	17.26	16.79	15.93	16.55	16.60	16.80
Carbohydrate intake (% of energy)	53.20	49.23	44.68	53.11	48.59	45.33

[a] All estimates accounted for complex survey designs. Values were mean for continuous variables and percentages for categorical variables. Abbreviation and acronyms: BMI body mass index..

Table S2. Associations between specific fatty acids and all-cause mortality and CVD mortality in the general population (n = 43450) [a]

	Tertiles of percentage energy from specific fatty acids			
	**Tertile 1**	**Tertile 2**	**Tertile 3**	***p*-Trend** [b]
SFA				
All-cause mortality				
MV-adjusted model[c]	1 (ref.)	1.02 (0.94-1.10)	1.08 (0.98-1.20)	0.09
CVD mortality				
MV-adjusted model[c]	1 (ref.)	0.85 (0.71-1.02)	0.86 (0.66-1.12)	0.35
MUFA				
All-cause mortality				
MV-adjusted model[c]	1 (ref.)	1.09 (0.93-1.28)	1.11 (0.90-1.37)	0.46
CVD mortality				
MV-adjusted model[c]	1 (ref.)	1.02 (0.85-1.22)	1.15 (0.87-1.51)	0.41
PUFA				
All-cause mortality				
MV-adjusted model[c]	1 (ref.)	0.96 (0.87-1.05)	0.92 (0.84-1.00)	0.20
CVD mortality				
MV-adjusted model[c]	1 (ref.)	0.93 (0.78-1.11)	0.88 (0.71-1.09)	0.33
ω-6 PUFA				
All-cause mortality				
MV-adjusted model[c]	1 (ref.)	0.96 (0.88-1.05)	0.93 (0.85-1.00)	0.05
CVD mortality				
MV-adjusted model[c]	1 (ref.)	0.89 (0.73-1.09)	0.89 (0.72-1.11)	0.27
LA				
All-cause mortality				
MV-adjusted model[c]	1 (ref.)	0.96 (0.88-1.05)	0.93 (0.86-1.01)	0.06
CVD mortality				
MV-adjusted model[c]	1 (ref.)	0.91 (0.76-1.09)	0.91 (0.74-1.13)	0.13
AA				
All-cause mortality				
MV-adjusted model[c]	1 (ref.)	0.94 (0.85-1.05)	0.90 (0.80-1.01)	0.06
CVD mortality				
MV-adjusted model[c]	1 (ref.)	0.87 (0.71-1.07)	0.87 (0.71-1.05)	0.34
ω-3 PUFA				
All-cause mortality				
MV-adjusted model[c]	1 (ref.)	0.96 (0.88-1.03)	0.90 (0.83-0.97)	0.01
CVD mortality				
MV-adjusted model[c]	1 (ref.)	0.84 (0.69-1.03)	0.80 (0.67-0.96)	0.02
ALA				
All-cause mortality				
MV-adjusted model[c]	1 (ref.)	0.99 (0.92-1.07)	0.97 (0.89-1.06)	0.45
CVD mortality				
MV-adjusted model[c]	1 (ref.)	0.88 (0.74-1.04)	0.91 (0.77-1.07)	0.27
Marine ω-3 PUFA				
All-cause mortality				
MV-adjusted model[c]	1 (ref.)	0.88 (0.79-0.97)	0.91(0.84-0.98)	0.03

CVD mortality				
MV-adjusted model[c]	1 (ref.)	0.56 (0.47-0.68)	0.69 (0.56-0.84)	0.003
DHA				
All-cause mortality				
MV-adjusted model[c]	1 (ref.)	0.90 (0.81-0.99)	0.91 (0.84-0.99)	0.04
CVD mortality				
MV-adjusted model[c]	1 (ref.)	0.54 (0.45-0.65)	0.70 (0.57-0.84)	0.003
EPA				
All-cause mortality				
MV-adjusted model[c]	1 (ref.)	0.90 (0.81-1.00)	0.87 (0.80-0.95)	0.003
CVD mortality				
MV-adjusted model[c]	1 (ref.)	0.51 (0.42-0.62)	0.62 (0.49-0.77)	0.001
DPA				
All-cause mortality				
MV-adjusted model[c]	1 (ref.)	0.79 (0.70-0.88)	0.87 (0.79-0.96)	0.01
CVD mortality				
MV-adjusted model[c]	1 (ref.)	0.54 (0.44-0.65)	0.57 (0.48-0.69)	<0.0001

[a] All estimates accounted for complex survey designs. [b] *p* trend was calculated by median values of each fatty acids. [c] Multivariable-adjusted model (MV-adjusted model) was adjusted for gender, age, race, educational levels, PIR, BMI, smoking status, alcohol drinking status, physical activity status, self-reported health status, baseline history of hypertension, hyperlipidemia, total energy intake, energy intake derived from carbohydrate, protein intake (in tertiles), cholesterol levels (in tertiles), and energy intake derived from remaining fatty acids (SFA, MUFA, and PUFA) where appropriate.

Table S3. Associations between specific fatty acids and all-cause mortality and CVD mortality after excluding patients who died within first two years of follow-up among patients with CMD (n = 7492) [a]

	Tertiles of percentage energy from specific fatty acids			
	**Tertile 1**	**Tertile 2**	**Tertile 3**	***p*-Trend** [b]
SFA				
All-cause mortality				
MV-adjusted model[c]	1(ref.)	0.93 (0.80-1.09)	0.99 (0.82-1.19)	0.96
CVD mortality				
MV-adjusted model[c]	1(ref.)	0.90 (0.62-1.31)	1.02 (0.68-1.55)	0.97
MUFA				
All-cause mortality				
MV-adjusted model[c]	1(ref.)	1.09 (0.93-1.28)	1.11 (0.90-1.37)	0.37
CVD mortality				
MV-adjusted model[c]	1(ref.)	0.92 (0.69-1.25)	1.00 (0.69-1.45)	0.90
PUFA				
All-cause mortality				
MV-adjusted model[c]	1(ref.)	0.93 (0.81-1.07)	0.86 (0.73-1.02)	0.06
CVD mortality				
MV-adjusted mode[lc]	1(ref.)	1.29 (1.03-1.63)	1.05 (0.76-1.47)	0.81
ω-6 PUFA				
All-cause mortality				
MV-adjusted model[c]	1(ref.)	0.92 (0.80-1.06)	0.88 (0.76-1.03)	0.08
CVD mortality				

MV-adjusted model[c]	1(ref.)	1.15 (0.88-1.49)	1.09 (0.77-1.55)	0.66
LA				
All-cause mortality				
MV-adjusted modelc	1(ref.)	0.93 (0.81-1.06)	0.88 (0.76-1.03)	0.08
CVD mortality				
MV-adjusted modelc	1(ref.)	1.13 (0.87-1.46)	1.08 (0.76-1.54)	0.73
AA				
All-cause mortality				
MV-adjusted model[c]	1(ref.)	0.98 (0.85-1.13)	1.06 (0.89-1.26)	0.50
CVD mortality				
MV-adjusted model[c]	1(ref.)	0.84 (0.59-1.17)	1.10 (0.77-1.58)	0.60
ω-3 PUFA				
All-cause mortality				
MV-adjusted model[c]	1(ref.)	1.02 (0.88-1.17)	0.89 (0.75-1.05)	0.15
CVD mortality				
MV-adjusted model[c]	1(ref.)	0.91 (0.71-1.17)	0.75 (0.53-1.06)	0.10
ALA				
All-cause mortality				
MV-adjusted model[c]	1(ref.)	1.00 (0.86-1.17)	0.96 (0.81-1.14)	0.69
CVD mortality				
MV-adjusted model[c]	1(ref.)	1.02 (0.80-1.31)	0.92 (0.64-1.32)	0.65
Marine ω-3 PUFA				
All-cause mortality				
MV-adjusted model[c]	1(ref.)	0.91 (0.78-1.07)	0.99 (0.85-1.15)	0.98
CVD mortality				
MV-adjusted model[c]	1(ref.)	0.69 (0.48-1.01)	0.85 (0.65-1.10)	0.31
DHA				
All-cause mortality				
MV-adjusted model[c]	1(ref.)	0.87 (0.74-1.02)	0.99 (0.86-1.15)	0.86
CVD mortality				
MV-adjusted model[c]	1(ref.)	0.54 (0.40-0.74)	0.88 (0.67-1.17)	0.72
EPA				
All-cause mortality				
MV-adjusted model[c]	1(ref.)	0.96 (0.80-1.14)	0.94 (0.81-1.09)	0.42
CVD mortality				
MV-adjusted model[c]	1(ref.)	0.58 (0.43-0.78)	0.62 (0.49-0.80)	0.001
DPA				
All-cause mortality				
MV-adjusted model[c]	1(ref.)	0.79 (0.69-0.91)	0.86 (0.75-0.99)	0.04
CVD mortality				
MV-adjusted model[c]	1(ref.)	0.64 (0.46-0.88)	0.65 (0.46-0.90)	0.01

[a] All estimates accounted for complex survey designs. [b] *p* trend was calculated by median values of each fatty acids. [c] Multivariable-adjusted model (MV-adjusted model) was adjusted for gender, age, race, educational levels, PIR, BMI, smoking status, alcohol drinking status, physical activity status, self-reported health status, baseline history of hypertension, hyperlipidemia, use of insulin, total energy intake, energy intake derived from carbohydrate, protein intake (in tertiles), cholesterol levels (in tertiles) and energy intake derived from remaining fatty acids(SFA, MUFA, and PUFA) where appropriate.

Table S4. Associations between specific fatty acids and all-cause mortality and CVD mortality after further adjustment of the use of medication (including diabete pills and insulin) (n = 8537) [a]

	Tertiles of percentage energy from specific fatty acids			
	**Tertile 1**	**Tertile 2**	**Tertile 3**	***P* trend**[b]
SFA				
All-cause mortality				
MV-adjusted model[c]	1(ref.)	1.00 (0.84-1.20)	0.96 (0.81-1.14)	0.69
CVD mortality				
MV-adjusted model[c]	1(ref.)	1.00 (0.67-1.50)	1.06 (0.61-1.85)	0.89
MUFA				
All-cause mortality				
MV-adjusted model[c]	1(ref.)	1.05 (0.91-1.21)	1.00 (0.80-1.25)	0.96
CVD mortality				
MV-adjusted model[c]	1(ref.)	1.06 (0.61-1.85)	0.98 (0.57-1.68)	0.92
PUFA				
All-cause mortality				
MV-adjusted model[c]	1(ref)	0.90 (0.77-1.05)	0.87 (0.74-1.03)	0.13
CVD mortality				
MV-adjusted mode[lc]	1(ref.)	1.52 (1.04-2.21)	1.10 (0.69-1.76)	0.63
ω-6 PUFA				
All-cause mortality				
MV-adjusted model[c]	1(ref.)	0.85 (0.73-0.99)	0.83 (0.70-0.97)	0.03
CVD mortality				
MV-adjusted model[c]	1(ref.)	1.16 (0.77-1.74)	1.00 (0.59-1.72)	0.90
LA				
All-cause mortality				
MV-adjusted modelc	1(ref.)	0.86 (0.74-1.00)	0.82 (0.69-0.97)	0.03
CVD mortality				
MV-adjusted modelc	1(ref.)	1.15 (0.77-1.72)	0.97 (0.56-1.68)	0.81
AA				
All-cause mortality				
MV-adjusted model[c]	1(ref.)	0.95 (0.84-1.07)	1.08 (0.91-1.30)	0.35
CVD mortality				
MV-adjusted model[c]	1(ref.)	0.97 (0.56-1.68)	1.13 (0.72-1.76)	0.56
ω-3 PUFA				
All-cause mortality				
MV-adjusted model[c]	1(ref.)	0.96 (0.82-1.12)	0.88 (0.74-1.05)	0.14
CVD mortality				
MV-adjusted model[c]	1(ref.)	0.95 (0.65-1.40)	0.74 (0.46-1.19)	0.20
ALA				
All-cause mortality				
MV-adjusted model[c]	1(ref.)	0.94 (0.80-1.10)	0.93 (0.77-1.12)	0.47
CVD mortality				
MV-adjusted model[c]	1(ref.)	0.81 (0.57-1.16)	0.97 (0.60-1.57)	0.97
Marine ω-3 PUFA				
All-cause mortality				
MV-adjusted model[c]	1(ref.)	0.87 (0.73-1.03)	0.93 (0.80-1.08)	0.56

CVD mortality				
MV-adjusted model[c]	1(ref.)	1.57 (0.98-2.52)	1.18 (0.71-1.97)	0.23
DHA				
All-cause mortality				
MV-adjusted model[c]	1(ref.)	0.89 (0.76-1.05)	0.92 (0.80-1.07)	0.43
CVD mortality				
MV-adjusted model[c]	1(ref.)	0.71 (0.44-1.14)	0.72 (0.47-1.09)	0.15
EPA				
All-cause mortality				
MV-adjusted model[c]	1(ref.)	0.95 (0.79-1.14)	0.91 (0.78-1.05)	0.21
CVD mortality				
MV-adjusted model[c]	1(ref.)	0.58 (0.40-0.86)	0.57 (0.39-0.84)	0.02
DPA				
All-cause mortality				
MV-adjusted model[c]	1(ref.)	0.80 (0.69-0.94)	0.86 (0.74-1.01)	0.11
CVD mortality				
MV-adjusted model[c]	1(ref.)	0.61 (0.38-1.00)	0.66 (0.43-1.00)	0.05

[a] All estimates accounted for complex survey designs. [b] *P* trend was calculated by median values of each fatty acids. [c] Multivariable-adjusted model (MV-adjusted model) was adjusted for gender, age, race, educational levels, PIR, BMI, smoking status, alcohol drinking status, physical activity status, self-reported health status, baseline history of hypertension, hyperlipidemia, use of medication (including diabete pills and insulin), total energy intake, energy intake derived from carbohydrate, protein intake (in tertiles), cholesterol levels (in tertiles) and energy intake derived from remaining fatty acids(SFA, MUFA, and PUFA) where appropriate.
